# The Association between Trajectories of Loneliness and Physical Frailty in Chinese Older Adults: Does Age Matter?

**DOI:** 10.3390/ijerph19095105

**Published:** 2022-04-22

**Authors:** Sha Sha, Sunny H. W. Chan, Lin Chen, Yuebin Xu, Yao Pan

**Affiliations:** 1 School of Sociology and Population Sciences, Nanjing University of Posts and Telecommunications, Nanjing 210023, China; shasha_1212@163.com; 2 School of Health and Social Wellbeing, University of the West of England, Bristol BS16 1DD, UK; sunny.chan@uwe.ac.uk; 3 Belt and Road School, Beijing Normal University at Zhuhai, Zhuhai 519087, China; linchen_988@163.com; 4 Institute of Advanced Studies in Humanities and Social Sciences, Beijing Normal University at Zhuhai, Zhuhai 519087, China; xuyuebin@bnu.edu.cn; 5 School of Social Development and Public Policy, Beijing Normal University, Beijing 100875, China

**Keywords:** loneliness, physical frailty, age difference, older adults

## Abstract

***Background*:** The present study aimed to examine age differences in the relationship between trajectories of loneliness and physical frailty among Chinese older adults. ***Methods*:** A total of 4618 participants aged ≥60 years old were taken from pooled data created from the 2011–2015 China Health and Retirement Longitudinal Study (CHARLS). Loneliness was assessed by a single question from the Centre for Epidemiological Studies scale, whereas physical frailty (PF) was examined by the physical frailty phenotype scale. We characterized trajectories of loneliness and PF using transition types and changes within the survey period. ***Results***: Logistic regression models revealed that baseline loneliness was significantly related to remaining robust PF (OR = 0.55, 95% CI = 0.49–0.63, *p* < 0.001) and worsening in PF (OR = 1.17, 95% CI = 1.05–1.30, *p* < 0.01) at follow-up. Baseline PF status was also significantly related to the transitions in loneliness (worsen: OR = 1.41, 95% CI = 1.11–1.78, *p* < 0.01; improve: OR = 0.65, 95% CI = 0.47–0.91, *p* < 0.05). The cross-lagged panel model found that baseline PF or loneliness had a significant predictive effect on the changes in each other. The associations between trajectories of loneliness and PF were weakened with age and clustered in the under 75 age groups. ***Conclusions:*** Bidirectional associations may exist between trajectories of loneliness and PF among Chinese older adults. Interventions should mainly target the young-old to reduce the adverse reciprocal effects of loneliness and PF.

## 1. Introduction

China is experiencing rapid social development, with life expectancy rising from 71.4 years at the turn of the century to 77.3 years in 2019 [1]. However, the average healthy life expectancy in 2018 was 68.7 years [2], which means that older adults may suffer from illness for more than eight years when approaching the end of their life. In addition, traditional family support for older adults in China is undergoing dramatic shocks due to the significant decline in fertility, uneven population mobility, and changing social attitudes. All of the above may contribute to the increased risks of experiencing loneliness and physical frailty among older adults.

Loneliness is a subjective feeling of dissatisfaction with social relationships [3], and has been a public health issue of global concern. It has become more prevalent, especially in the context of the social restrictions imposed around the world under the COVID-19 pandemic [4,5]. In recent years, the trajectory of loneliness has tended to be established in several studies [6], developing the understanding of the variability of its state and extent in older adults. Although research has proven the association between loneliness and fatal physical health outcomes [7,8,9], little is known about the relationship between the trajectory of loneliness, i.e., loneliness transition types and changes, and adverse health-related outcome.

Physical frailty, an age-related syndrome, is recognized as an increased vulnerability and decreased capability of physiological reversal [10,11]. It leads to more challenges to social care and health systems worldwide. For instance, recent studies indicated that frail older adults tend to be more susceptible to COVID-19 and frailty has negative effects on the prognosis of COVID-19 patients [12,13]. In this light, it is worthwhile to gain better insights into the risk factors of the trajectory of physical frailty since previous studies have found that the process of physical frailty development is dynamic and reversible [14,15,16].

### 1.1. The Relationship between Loneliness and Physical Frailty

Some longitudinal studies found that a higher degree of loneliness at baseline predicted higher risks of occurrence of physical frailty [17]; for example, among community-dwelling older adults over 60 years old in Singapore, those who felt lonely were associated with higher levels of frailty [18]. Alternatively, some studies showed that frail older adults tended to have a higher level of loneliness than non-frail adults [19,20,21] and baseline physical frailty even predicted an increase in loneliness at follow-up [22]. These may imply a potential bidirectional relationship between loneliness and physical frailty.

Although there is growing evidence of the relationship between loneliness and frailty, trajectory relationships regarding the transition types and changes of the two remain scarce. Previous studies have found that higher levels of loneliness increased the risk of worsening in physical frailty and decreased the likelihood of recovery from frailty in older adults [23,24,25], but none have specified the relationship between physical frailty and loneliness transitions. Given the erratic nature and devastating health impacts of loneliness and physical frailty, examining the relationship between the trajectories of loneliness and physical frailty may be helpful to gain a full picture and have valuable policy implications for the public health systems.

### 1.2. Age Role in the Relationship between Loneliness and Physical Frailty

Older adults are heterogeneous. Different age-related conditions may play different roles as older adults adapt to age-related changes [26]. The relationship between loneliness and physical frailty may also be complicated by age, as old-old (usually ≥80 years old) may gain positive influences from age. For example, a previous study found frailty was less likely associated with depressive symptoms when people aged [27].

A paradox of aging has been proposed, namely, that declines in physical function and strength tend to be accompanied by advancing age, but the adverse effects do not necessarily increase [28,29]. These suggest a protective effect of age in the relationship between well-being and physical health. Furthermore, the age difference in the relationship between loneliness and physical frailty may help to provide evidence for age-targeted intervention strategy.

Therefore, the present study aimed to investigate the association between trajectories of loneliness and physical frailty in different age groups of older adults. There are two hypotheses: (1) the relationships between the trajectory of loneliness and frailty are bidirectional; and (2) the relationships between the trajectory of loneliness and frailty might be weaker in the older age group. We employed a longitudinal design with a set of nationally representative data to address this issue. Moreover, to extend previous studies, we characterized transition types and changes to assess the trajectories of the relationship between loneliness and physical frailty.

## 2. Materials and Methods

### 2.1. Date Source and Participants

The China Health and Retirement Longitudinal Study (CHARLS) is a nationally representative dataset of Chinese households and individuals aged over 45 years old [30]. This national study started in 2011 and was followed up every 2–3 years. The protocols were approved by the Ethics Review Committee at Peking University. Participants signed informed consent forms before joining the study. The CHARLS utilized a multistage stratified probability-proportionate-to-size sampling design, covering 150 county-level units and 450 village-level units [31]. Details on the survey design and data collation for CHARLS have been described elsewhere before [30,32] and on the official website http://charls.pku.edu.cn/ (accessed on 31 March 2022). Data from CHARLS is publicly available. Formal approval from an institutional review board was unnecessary in the present study.

For the current analysis, we used three waves of CHARLS from 2011 to 2015, because the latest 2018 survey data did not provide sufficient information to measure physical frailty in older adults. A total of 17,596 community residents aged 45 years and above participated in the CHARLS baseline survey (2011 survey, wave 1), followed by 18,455 participants in the 2013 survey (wave 2) and 20,967 participants in the 2015 survey (wave 3). We created a pooled dataset that included only participants who were aged 60 years or above in 2011 and participated in any two waves of the survey between 2011 and 2015. The pooled data contained four cohorts: (1) cohort of participants in both 2011 and 2013 waves (cohort 2011–13); (2) cohort of participants in both 2013 and 2015 waves (cohort 2013–15); (3) cohort of participants in both 2011 and 2015 waves (cohort 2011–15); (4) cohort of participants in each of 2011, 2013 and 2015 waves (cohort 2011–13–15). In each cohort, the first wave served as baseline (T_1_) and the last wave as follow-up (T_2_). We also created a variable indicating the cohort.

A total of 7546 older adults were included in pooled data. Of those, 4618 (61.2%) participants who had data on measures of loneliness and physical frailty in each cohort were selected for further analysis in this study. Table S1 summarizes the characteristics of participants in different age groups at baseline. It can be seen that 40% of the participants are in the 60–64 age group (60–64 years old), 46% in the 65–74 age group (65–74 years old), and 14% in ≥75 age group (≥75 years old). Significant differences were found among older adults of different age groups. Compared to the younger elderly, the older elderly tended to be single, male, less educated, with higher income, comprise fewer smokers, have less contact with children, and have worse cognitive ability.

### 2.2. Measures

#### 2.2.1. Trajectory of Loneliness

Loneliness was measured by a widely used single question from the Centre for Epidemiological Studies scale (CES-D), asking participants how often they felt lonely during the last week. The single-question measure of loneliness has been shown to be valid and appropriate for assessing the aging population [33] and has been used in previous studies to analyze the transitions of loneliness [8,34,35]. We classified the four-point response scale “rarely or none of the time” as a low level of loneliness, “Some or a little of the time” or “Occasionally or a moderate amount of the time” as a medium level of loneliness, and “most or all of the time” as a high level of loneliness.

The trajectory in loneliness was defined in two ways: (1) transitions in loneliness: maintain, when loneliness levels were the same levels in T_1_ and T_2_; worsen, when loneliness transferred to higher levels in T_2_ compared to T_1_; improve, when loneliness levels were lower in T_2_ than T_1_; and (2) changes in loneliness: the difference in loneliness between T_2_ and T_1_.

#### 2.2.2. Trajectory of Physical Frailty

Physical frailty (PF) was operationalized by the most widely used and validated physical frailty phenotype (PFP) scale, which includes five elements: weakness, slowness, exhaustion, low activity, and shrinking [36]. Although, due to the standard activity criteria design, only half of the randomized participants took part in the survey module, previous studies have demonstrated the validity of the measurement of physical frailty in CHARLS data [37,38,39,40]. Details on each of the five criteria are provided in Table S2.

We measured trajectory in PF in two ways. Firstly, four physical frailty transition types were designed: remain healthy, indicating that physical frailty status was robust in T_1_ and T_2_; worsen, indicating a change from robust to prefrail or prefrail to frail between T_1_ and T_2_; improve, meaning that physical frailty status was transferred from prefrail to robust, frail to prefrail, or frail to robust between T_1_ and T_2_; and remain unhealthy, indicating that participants were in prefrail or frail in both T_1_ and T_2_. Secondly, change in physical frailty was constructed as the difference between the physical frailty measures results at T_2_ and T_1_.

#### 2.2.3. Age and Covariates

Age was categorized into three age groups: 60–64 age group (60–64 years old), 65–74 age group (65–74 years old), and ≥75 age group (≥75 years old).

Sociodemographic information included in this study were gender (male or female), residence (urban or rural), education level (illiterate, no formal education, elementary school, middle school, or above), marital status (without a spouse or with a spouse), frequency of contact with children (seldom contact, monthly contact, weekly contact), and income. Medical information contained self-reported health (good, so so, bad), number of chronic diseases, and smoking (no or yes). Other covariates included activity participation frequency and cognitive ability. Activity participation frequency was summed from the frequency of participants’ participation in 10 activities in the previous month. Each activity frequency scored from 0 (not participated) to 3 (almost every day). Based on previous studies [41,42], cognitive ability was measured by episodic memory and mental intactness. Episodic memory was assessed by the ability to recall, either immediately and after a delay, ten Chinese words. Mental intactness was derived from subscales of the Telephone Interview of Cognitive Status, which included orientation, numerical ability, and drawing ability. The total score of cognitive ability was 31, with higher scores indicating better cognitive ability.

### 2.3. Statistical Analysis

Descriptive statistics at baseline were summarized using means (±standard deviation) or counts (percentages). The chi-square test was applied to compare the baseline characteristics among age groups.

The model analysis was performed in two parts. In stage I, two logistic regression models were used to estimate odds ratios (ORs) and confidence intervals (CIs) for the whole sample and different age groups of older adults: (a) the effect of baseline levels of loneliness on transition types of PF; and (b) the effect of baseline levels of PF on transition types of loneliness.

**Figure 1:**
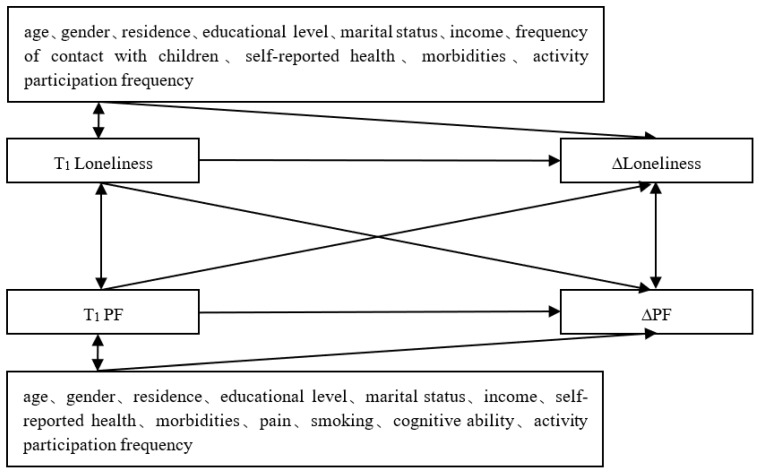
Conceptual diagram of cross-lagged associations between change in loneliness and PF. ∆Loneliness = T_2_ Loneliness − T_1_ Loneliness; ∆PF = T_2_ PF − T_1_ PF.

In stage II, a cross-lagged panel model (CLPM) was utilized to examine the association between changes in loneliness and PF in the total sample and different age groups. CLPM is an analytical instrument used to describe the potential reciprocal relationships or directional effects between variables over time [43]. Within the model system, we tried to focus on differences across age groups in: (a) the cross-lagged effect of baseline loneliness on the change in PF; and (b) the cross-lagged effect of baseline PF on the change in loneliness. The schematic diagram of the model is shown in Figure 1.

We used the full information maximum likelihood method (FIML) to handle missing data and the robust estimator (MLR) to handle non-normal distributions of the data. As is common in cross-lagged analysis, goodness-of-fit indices include the comparative fit index (CFI), root mean square error of approximation (RMSEA), and standardized root means square residual (SRMR). Models with CFI values >0.90 are considered to have acceptable fit and >0.95 good fit, and models with RMSEA and SRMR values <0.08 indicate acceptable fit and <0.05 good fit (Bentler and Bonett, 1980; Hu and Bentler, 1999). The influences of loneliness and PF were not identical; we used different covariates in the model with loneliness as the dependent variable and the model with PF as the dependent variable.

Based on the proportion of older adults in age groups, we repeated analyses using different cutoff values of age to conduct additional sensitivity analyses. We performed two additional groupings of participant ages: 60–64 years old vs. 65–69 years old vs. ≥70 years old; 60–64 years old vs. 65–79 years old vs. ≥80 years old. Stata 15.0 (Stata Statistical Software Release 15, StataCorp., College Station, TX, USA) was used for basic statistical analysis. Mplus 8.0 (Version 8.0, Muthén & Muthén, Los Angeles, CA, USA) was used for CLPM analysis.

## 3. Results

### 3.1. Associations between Baseline Loneliness and PF Transitions as Well as Baseline PF and Loneliness Transitions in Older Adults

A full description of the transition types of loneliness and PF can be found in Table S3. The associations between baseline loneliness and PF transitions, and between baseline PF and loneliness transitions, are shown in Table 1.

In the total sample, baseline loneliness levels reduced the likelihood of older adults to maintain baseline robust status, suggesting an increased risk of PF at follow-up (OR = 0.55, 95% CI = 0.49–0.63, *p* < 0.001) and also increased the risk of worsening PF in older adults at follow-up (OR = 1.17, 95% CI = 1.05–1.30, *p* < 0.01). Furthermore, PF status at baseline had a significant effect on the level of loneliness in older adults at follow-up. Older adults who were in prefrail status at baseline had a higher risk of worsening loneliness levels at follow-up (OR = 1.41, 95% CI = 1.11–1.78, *p* < 0.01) and a lower likelihood of experiencing loneliness improvement (OR = 0.65, 95% CI = 0.47–0.91, *p* < 0.05). Hence, a two-way association between loneliness and PF in terms of baseline transition types was implied.

We also found such associations were significant only in the 60–64 and 64–74 age groups, whereas the significant effect was absent in older adults in the ≥75 age group. In Model 1, the baseline loneliness reduced the likelihood of robust PF in the 60–64 years group (OR = 0.50, 95% CI = 0.41–0.61, *p* < 0.001) and the 64–74 years group (OR = 0.60, 95% CI = 0.49–0.72, *p* < 0.001). High levels of baseline loneliness also increased the risk of worsening PF in these two groups (60–64 years group: OR = 1.26, 95% CI = 1.05–1.52, *p* < 0.05; 64–74 years group: OR = 1.21, 95% CI = 1.03–1.42, *p* < 0.05). Model 2 showed a higher risk of worsening loneliness in baseline frail older adults who are in the 60–64 years age group (OR = 3.64, 95% CI = 1.25–10.56, *p* < 0.05) and the 64–74 years age group (OR = 1.47, 95% CI = 1.05–2.07, *p* < 0.05). It further indicated that the robust two-way relationship between loneliness and PF in terms of baseline and transition types was only found in those older adults who were under 75 years old.

### 3.2. Stage II: Associations between Loneliness and PF Changes in Older Adults

**Figure 2:**
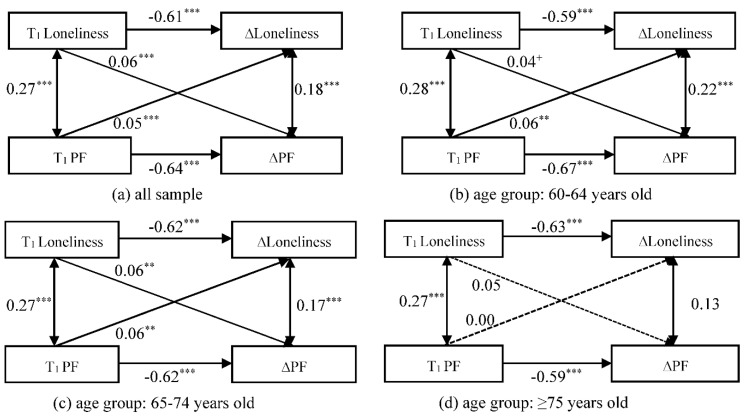
Standardized estimates of the cross-lagged relationship between change in loneliness and change in PF. *** *p* < 0.001, ** *p* < 0.01, * *p* < 0.05, ^+^ *p* < 0.1.

Figure 2 provides the standardized estimates of the CLPM models for the associations between changes in loneliness and PF for different age groups of older adults throughout the survey period. The fit indices for the CLPM model with the total sample were CFI = 0.999, RMSEA = 0.015, and SRMR = 0.003. The fit indices for the CLPM model with the subgroup were CFI = 1.000, RMSEA = 0.011, and SRMR = 0.003.

In the correlation paths, there were significant positive correlations between loneliness and PF in the total sample and different age groups. This indicates that, at the same time point, older adults with high levels of loneliness tend to have more severe levels of PF. The changes in loneliness and PF were found to have a statistically significant correlation only in the age groups under 75 years old. This suggests that, at the same time point, young-old with worsening PF were likely to be accompanied by deepening levels of loneliness.

In the cross-lagged paths, we found both baseline loneliness and PF had a significant positive predictive effect on the changes in each other (T_1_ Loneliness → ∆PF: β = 0.06, *p* < 0.001; T_1_ PF → ∆Loneliness: β = 0.05, *p* < 0.001). This suggests a potential bidirectional association between changes in loneliness and PF in the total sample. In particular, similar bidirectional associations were found in the 60–64 and 65–74 age groups. In the ≥75 age group, the effect of baseline loneliness or PF on the change in PF or loneliness was no longer significant, however. Specifically, baseline PF had significant positive cross-lagged effects on the change in loneliness in the age groups of 60–64 (β = 0.06, *p* < 0.01) and 65–74 (β = 0.06, *p* < 0.01). This implies that young-old with higher baseline PF may deepen their loneliness level in the subsequent time period. Furthermore, there was a positive cross-lagged association between baseline loneliness and PF change in the age groups of 60–64 (β = 0.04, *p* = 0.057) and 65–74 (β = 0.06, *p* < 0.001). This suggests that the young-old with higher loneliness at baseline are also more likely to have increased levels of PF in the future.

### 3.3. Sensitivity Analyses

In the sensitivity analyses with an age threshold of 70 years, 40% of the participants were in the 60–64 age group (60–64 years old), 28% in the 65–69 age group (65–69 years old), and 32% in ≥70 age group (≥70 years old) (Tables S4 and S5). The effect of baseline loneliness or PF on the type of transition status and the relationship between changes in loneliness and PF were not exclusively clustered in young-old (60–64 and 65–69 age groups). In the ≥70 age group, higher levels of baseline loneliness also significantly reduced the probability of maintaining baseline PF status at follow-up (OR = 0.61, 95% CI = 0.47–0.68, *p* < 0.001). Among older adults in the ≥70 age group, those who were in prefrail state at baseline had an increased risk of worsening loneliness and a decreased likelihood of improving loneliness at follow-up (worsen loneliness: OR = 1.52, 95% CI = 1.01–2.28, *p* < 0.05; improve loneliness: OR = 0.51, 95% CI = 0.29–0.90, *p* < 0.05). Among those aged ≥70 years, baseline loneliness was a significant predictor of change in PF (β = 0.08, *p* < 0.01), and baseline PF positively predicted change in loneliness at a 10% significance level (β = 0.04, *p* = 0.071).

In addition, in the sensitivity analyses with an age threshold of 80 years, 40% of the participants were in the 60–64 group (60–64 years old), 56% in the 65–79 group (65–79 years old), and 4% in the ≥80 group (≥80 years old) (Tables S4 and S5). The effects of baseline loneliness or PF on transition state type and the interrelationships between changes in loneliness and PF in the older age group (≥80 years old) were similar to those with an age threshold of 75 years. In the ≥80 age group, baseline loneliness levels or baseline PF did not show a significant effect on the transition types of change in PF or loneliness, and there was no significant cross-lagged relationship between the effects of loneliness and PF on changes in each other.

## 4. Discussion

The present study aimed to extend the current understanding of the association between loneliness and PF. To do so, transition types and changes in loneliness and PF between baseline and following waves were computed and categorized to characterize the trajectories of loneliness and PF. We employed different models in age groups to examine the associations in pooled data from a nationally representative survey of China.

As expected, we found the reciprocal effect between loneliness and PF in terms of baseline and transition types. The finding is consistent with some previous studies showing that higher levels of loneliness associated with PF or PF transition types [17,22]. In addition, we found significant predictive associations between baseline loneliness and the subsequent changes in PF, and baseline PF and the following variations in loneliness. These findings supported our first hypothesis and imply a bidirectional relationship between trajectories of loneliness and PF.

The second objective of our study was to examine the age difference in the association between trajectories of loneliness and PF. Consistent with our second hypothesis, we found that the significant effects of baseline loneliness or PF on the transitions of each other were clustered only in the age groups under 75 years old. Specifically, for the young-old, their higher baseline PF levels significantly predicted the changes in the subsequent levels of loneliness. Alternatively, the baseline loneliness also had projecting effects on changes in PF of these two age groups. Nevertheless, none of these relations were significant in the older adults of the ≥75 years age group. These findings provide evidence of the protective effect of age on the associations of trajectories of loneliness and PF, and correspond to the paradox of the aging phenomenon.

There are two potential explanations for the age paradox in our findings. Firstly, Socioemotional Selectivity Theory suggests that one’s perception of survival time plays a crucial role in choosing and pursuing social goals [44,45]. Compared to young-old counterparts, old-old are more aware of the finite nature of time. Hence, they are more motivated to regulate social goals and emotional experiences to amplify positive emotions and reduce negative emotions. This is also demonstrated by brain science research emphasizing the fact that age-related shifts in preferred strategies and priorities have important influences on understanding the emotional well-being of older adults [46]. It thus helps to explain the smaller impact of loneliness on those people in the ≥75 age group. Secondly, previous studies also found that the association between PF and depression is stronger in the younger age group [27], with PF having a more negative impact on life satisfaction in the younger than in the old age group [47]. This may be due to the gaining of experience in the aging process, so that the old-old can cope effectively with aging-related problems, and are thus more likely to harness the corresponding poor health conditions (e.g., PF) that accompany aging [48,49].

Given the significant evidence of the mutual impact of loneliness and frailty, the recognition of the role of the health care system in addressing loneliness is important. Older adults, especially the frail elderly, are particularly numerous and frequent users of the health care system. Clinicians or physicians can pay more attention to the “loneliness” factor when coming into contact with this client group. Early intervention by referring elderly to proper services, such as community service centers, cannot only protect them from worsening outcomes, but also decrease the use of inpatient care and reduce health care provider visits.

Furthermore, while confirming the robustness of the results, sensitivity analyses also suggest a noteworthy threshold issue regarding the age paradox. Previous studies have typically used >80 years old as the cutoff for the older age group when discussing the role of age [47,50]. We found that although there was no significant association between the trajectories of loneliness and PF for the ≥80 age group, the protective effect of age may have commenced as early as 75 years old. The reasons for this discrepancy may be due to the different variable relationships and samples utilized, and more research is needed to validate this. However, the significant correlation between loneliness and PF at baseline in the old-old age group (≥75 or ≥80) suggests that old-old may still suffer from loneliness and PF, and the cross-lagged effect between the two was no longer significant. This may even make it more difficult to help old-old as problems with loneliness or PF may separately develop or worsen. This also implies the importance of early screening and the efficiency of identifying target populations for loneliness or PF health policy interventions. For example, annual physical examinations in China are provided free of charge to older adults, and have high participation rates [51]. Community service centers and primary health workers can also take advantage of this opportunity to conduct focused screening for physical health (PF) and mental health (loneliness) for those under the age of 75 at the community level. In particular, it may help in advance to avoid the establishment of a vicious circle by observing another health domain when one is found to be impaired.

The strengths of our study include the new perspective on the relationship between loneliness and PF and the representative national sample of China. The study also has limitations. First, a single-question measure of loneliness may underestimate its impact [52], and it may lead to problems in measuring changes in loneliness. Future research may expand the role of loneliness on physical health by using a multidimensional scale. Second, since CHARLS was designed to collect high-quality data representative of middle-aged and older Chinese adults, and the PF measurement operation is more difficult for older adults, the analytic sample we used had a younger mean age (67.0 years) and a lower proportion of participants aged 75 years and above (14%). This may have biased the results. However, when we used 70 years old as a threshold in sensitivity analysis, the proportion of the sample aged 70 and above was also less than one-third. The sensitivity analysis results confirmed a significant relationship between the trajectory of loneliness and PF in the ≥70 age group. This yielded differences from the analysis of the ≥75 age group, which together suggest a role of age. Third, more psychological factors, such as depressive symptoms, were not controlled for in this study. Future research could expand our understanding of the relationship between loneliness and PF and the role of age with other representative data and multidimensional loneliness scales.

## 5. Conclusions

The present study attempted to extend the understanding of the association between loneliness and PF. The findings suggest a potential bidirectional association between trajectories of loneliness and PF among a nationally representative sample of older Chinese adults. The results illustrate the long-term nature of the relationship and provide longitudinal research evidence for the design of intervention strategies for loneliness and PF. In addition, the relationship between trajectories of loneliness and PF was found to be mainly focused on young-old (<75 years old), which may contribute to the focus of the policy intervention population and imply the need for early interventions for loneliness and PF.

## Figures and Tables

**Table 1:** Odds ratios (95% CI) for baseline loneliness and PF transition types, and baseline PF and loneliness transition types.

	Model 1: PF Transition Types (OR (95% CI))				Model 2: Loneliness Transition Types (OR (95% CI))		
	Remain Robust	Worsen	Improve	Remain Unhealthy	Maintain	Worsen	Improve
Baseline loneliness							
total	0.55 ***	1.17 **	0.94	1.11 *			
	[0.49–0.63]	[1.05–1.30]	[0.85–1.04]	[1.02–1.21]			
60–64	0.50 ***	1.26 *	0.93	1.14			
	[0.41–0.61]	[1.05–1.52]	[0.79–1.10]	[0.98–1.32]			
65–74	0.60 ***	1.21 *	0.92	1.08			
	[0.49–0.72]	[1.03–1.42]	[0.79–1.07]	[0.95–1.24]			
≥75	0.67	0.97	0.98	1.07			
	[0.44–1.01]	[0.74–1.27]	[0.73–1.32]	[0.86–1.34]			
Baseline PF							
total							
prefrail					0.91	1.41 **	0.65 *
					[0.75–1.11]	[1.11–1.78]	[0.47–0.91]
frail					0.76	1.56	0.83
					[0.48–1.18]	[0.90–2.72]	[0.45–1.54]
60–64							
prefrail					1.01	1.25	0.59
					[0.72–1.41]	[0.84–1.87]	[0.32–1.07]
frail					0.48	3.64 *	0.45
					[0.19–1.18]	[1.25–10.56]	[0.08–2.40]
65–74							
prefrail					0.81	1.47 *	0.78
					[0.62–1.08]	[1.05–2.07]	[0.50–1.22]
frail					0.96	1.11	0.81
					[0.49–1.85]	[0.49–2.54]	[0.34–1.89]
≥75							
prefrail					1.07	1.39	0.45
					[0.64–1.78]	[0.75–2.60]	[0.20–1.01]
frail					0.82	0.95	1.11
					[0.33–2.05]	[0.22–4.19]	[0.32–3.90]

Note: *** *p* < 0.001, ** *p* < 0.01, * *p* < 0.05. The model had been adjusted for all covariates. Model 1 was adjusted for the component numbers in the PFP scale at baseline and Model 2 was adjusted for the baseline levels of loneliness.

## Data Availability

The datasets are publicly available from the project of the China Health and Retirement Longitudinal Study (CHARLS) and can be downloaded after registration from: http://charls.pku.edu.cn/ (accessed 31 March 2022).

## Supplementary Materials

The following supporting information can be downloaded at: https://www.mdpi.com/article/10.3390/ijerph19095105/s1. Table S1: Baseline characteristics of the participants according to different age groups. Table S2: Definition and values of five criteria for physical frailty in older adults. Table S3: Physical frailty and loneliness transition types between baseline and follow-up. Table S4: Odds ratios (95% CI) for baseline loneliness and PF transition types, baseline PF and loneliness transition types. Table S5: Standardized estimates of the cross-lagged relationship between change in loneliness and change in physical frailty.
