# Supplementary material for: The Association between Trajectories of Loneliness and Physical Frailty in Chinese Older Adults: Does Age Matter?
Source: Int J Environ Res Public Health. 2022 Apr 22;19(9):5105. doi: 10.3390/ijerph19095105 (PMC9101367; doi:10.3390/ijerph19095105)
Supplement: Supplementary file 1 [file ijerph-19-05105-s001.zip › ijerph-1637416-supplementary.pdf]

Supplementary materials

Table S1. Baseline characteristics of the participants according to different age groups

	60-64 (N =1849)	65-74 (N =2141)	≥75 (N =628)	Total (N =4618)	P-value
gender, n(%)					0.025
women	931(50.4)	986(46.1)	301(47.9)	2218(48.0)	
men	918(49.6)	1155(53.9)	327(52.1)	2400(52.0)	
residence, n(%)					0.065
rural	1218(65.9)	1406(65.7)	383(61.0)	3007(65.1)	
urban	631(34.1)	735(34.3)	245(39.0)	1611(34.9)	
education level, n(%)					<0.001
illiterate	512(27.7)	670(31.3)	344(54.8)	1526(33.1)	
no formal education	471(25.5)	421(19.7)	122(19.4)	1014(22.0)	
elementary school	525(28.4)	624(29.2)	92(14.6)	1241(26.9)	
middle school or above	340(18.4)	425(19.9)	70(11.1)	835(18.1)	
marital status, n(%)					<0.001
without spouse	155(8.4)	378(17.7)	232(36.9)	765(16.6)	
with spouse	1694(91.6)	1763(82.3)	396(63.1)	3853(83.4)	
income, mean(SD)	4981.7 (10646.4)	6080.7 (11750.6)	6460.1 (12021.3)	5690.8 (11371.4)	0.002
self-rated health, n(%)					0.011
good	388(21.0)	408(19.1)	153(24.4)	949(20.6)	
so so	879(47.6)	1003(46.8)	298(47.5)	2180(47.2)	
bad	580(31.4)	730(34.1)	177(28.2)	1487(32.2)	
number of chronic diseases, mean(SD)	1.5(1.4)	1.6(1.4)	1.4(1.4)	1.5(1.4)	0.024
pain, n(%)					0.239
no	1152(64.8)	1290(65.2)	399(68.6)	2841(65.5)	
yes	625(35.2)	690(34.8)	183(31.4)	1498(34.5)	
smoking, n(%)					0.012
no	1077(64.3)	1209(63.2)	382(70.1)	2668(64.6)	
yes	598(35.7)	704(36.8)	163(29.9)	1465(35.4)	
frequency of contact with children, n(%)					<0.001
seldom contact	191 (19.4)	333 (24.5)	186 (40.4)	710 (25.3)	
monthly contact	240 (24.3)	364 (26.8)	66 (14.3)	670 (23.9)	
weekly contact	555 (56.3)	663 (48.8)	208 (45.2)	1426 (50.8)	
activity participation frequency, mean(SD)	1.5(2.0)	1.5(2.0)	1.6 (1.9)	1.5(2.0)	0.290
cognitive ability, mean(SD)	13.7(5.5)	12.5(5.6)	10.0(5.6)	12.6(5.7)	<0.001

Table S2. Definition and values of five criteria for physical frailty in older adults

Weakness was measured using a hand-held mechanical dynamometer by maximum grip strength of either hand (two tests for each hand, standing position). Adjusting for gender and body mass index (BMI), those ≤ 20th percentiles of the participants' distribution were defined as weakness. Slowness was measured by walking speed, using the average time taken in two trials of 2.5 meters. Adjusting for gender and height, those ≤ 20th percentile of the participants' distribution was defined as slowness. Exhaustion was represented by two questions from the Center for Epidemiological Studies Depression (CESD) scale. Participants met criteria of exhaustion if they answered "Occasionally or a moderate amount of the time (3-4 days)" or "Most or all of the time (5-7 days)" to either of the two questions: "I felt everything I did was an effort" and "I could not get going." Participants were defined as a low activity if they did not make any vigorous activities/moderate physical effort/ walking for at least 10 minutes continuously during a week. Participants met shrinking criteria if they were self-reporting loss of 5 or more kilograms between wave 1 and wave 2 or declined 5 or more kilograms between wave 2 and wave 3.

PF was used as categorial variable when we constructed physical frailty status transition types. Participants with no criteria were classified as robust, with one or two criteria were classified into prefrail, and those with three to five criteria were defined as frail. The PF was used as continuous variable in the operation of changes in PF. The number of criteria identified physical frailty level met, with more standards indicating high levels of physical frailty.

Criteria	Definition	Values	
		Female	Male
weakness	gait strength	BMI≤20.6kg/m^2:≤18.0kg	BMI≤20.0kg/m^2:≤27.0kg
		20.6<BMI≤23.1kg/m^2:≤18.2kg	20.0<BMI≤22.0kg/m^2:≤28.5kg
		23.1<BMI≤25.7kg/m^2:≤20.0kg	22.0<BMI≤24.4kg/m^2:≤30.0kg
		BMI>25.7kg/m^2:≤20.0kg	BMI>24.4kg/m^2: ≤31.5kg
walking	walking speed	height≤151cm:≤0.41m/s<br>height >151cm: ≤0.45m/s	height≤162cm:≤0.47m/s<br>height >162cm:≤0.50m/s
exhaustion	self-reported	felt " everything I did was an effort " or " I could not get going " about 3-4 days or 5-7 days in a week	
low activity	self-reported	did not do any vigorous activities or moderate activities or walking for at least 10 min continuously during a usual week	
shrinking	self-reported	lost 5 or more kilograms in the last year or weight declined 5 or more kilograms between wave 2 and wave 3.	

BMI: body mass index

Table S3. Physical frailty and loneliness transition types between baseline and follow-up

Table 3 shows the transition types of physical frailty and loneliness between the baseline and follow-up.

Of the 4618 participants at baseline, 1097 (23.8%) participants experienced worsening physical frailty and 894 (19.4%) participants transitioned to a better physical frailty status at T_2. Nearly 22% (1025) of participants remained robust and 35% (1602) participants continued in an unhealthy state (prefrail or frail) from baseline to T_2. There were significant differences in the distribution of physical frailty transition types between age groups. The proportion of people under 65 years of age who maintained baseline robust status and experienced improvement was higher than those in the other two groups. Nearly 30% of participants over age of 75 experience worsening in physical frailty status and over 40% maintained their baseline unhealthy physical frailty status.

Most of participants maintained their baseline loneliness levels across the waves. The proportion of the baseline older adults with worsening loneliness and improving loneliness was 17.6% and 18.0%, respectively. The type of loneliness transitions was significantly different between T_1 and T_2 for participants in different age groups. ⩾75 group had a lower rate of maintaining baseline loneliness status and a higher rate of improvement than the younger older group.

	T1-T2			
	total(n, %)	60-64(n, %)	65-74(n, %)	⩾75(n, %)
physical frailty				
remain robust	1025(22.2)	494(26.7)	454(21.2)	77(12.3)
worsen	1097(23.8)	395(21.4)	514(24.0)	188(29.9)
improve	894(19.4)	397(21.5)	399(18.6)	98(15.6)
remain unhealthy	1602(34.7)	563(30.5)	774(36.2)	265(42.2)
loneliness				
maintain	2973(64.4)	1213(65.6)	1375(64.2)	385(61.3)
worsen	813(17.6)	336(18.2)	371(17.3)	106(16.9)
improve	832(18.0)	300(16.2)	395(18.5)	137(21.8)

Notes: chi-squared test for physical frailty transitions by age groups between T1 and T2 : $p < 0.0001$;
chi-squared test for loneliness transitions by age groups between T1 and T2 : $p < 0.032$;

Table S4. Odds ratios (95% CI) for baseline loneliness and PF transition types, baseline PF and loneliness transition types

	Model 1: PF transition types (OR(95%CI))				Model 2: loneliness transition types (OR(95%CI))		
	Remain robust	Worsen	Improve	Remain unhealthy	Maintain	Worsen	Improve
baseline loneliness							
60-64	0.50***	1.26*	0.93	1.14			
	[0.41 - 0.61]	[1.05 - 1.52]	[0.79 - 1.10]	[0.98 - 1.32]			
65-70	0.60***	1.18	0.99	1.07			
	[0.48 - 0.76]	[0.97 - 1.45]	[0.82 - 1.20]	[0.90 - 1.27]			
⩾70	0.61***	1.01	0.87	1.12			
	[0.47 - 0.78]	[0.91 - 1.34]	[0.71 - 1.05]	[0.96 - 1.30]			
60-64	0.50***	1.26*	0.93	1.14			
	[0.41 - 0.61]	[1.05 - 1.52]	[0.79 - 1.01]	[0.98 - 1.32]			
65-80	0.60***	1.16*	0.93	1.09			
	[0.50 - 0.71]	[1.01 - 1.34]	[0.81 - 1.07]	[0.97 - 1.22]			
⩾80	0.91	1.01	0.75	1.06			
	[0.49 - 1.69]	[0.66 - 1.84]	[0.43 - 1.32]	[0.72 - 1.56]			
baseline PF							
60-64							
prefrail					1.01	1.25	0.59
					[0.72 - 1.41]	[0.84 - 1.87]	[0.32 - 1.07]
frail					0.48	3.64*	0.45
					[0.19 - 1.18]	[1.25 - 10.56]	[0.08 - 2.40]
65-70							
prefrail					0.80	1.41	0.89
					[0.56 - 1.14]	[0.92 - 2.16]	[0.52 - 1.55]
frail					0.81	0.91	1.37
					[0.29 - 2.27]	[0.23 - 3.57]	[0.41 - 4.62]
⩾70							
prefrail					0.92	1.52*	0.51*
					[0.66 - 1.30]	[1.01 - 2.28]	[0.29 - 0.90]
frail					0.88	1.19	0.80
					[0.46 - 1.67]	[0.51 - 2.75]	[0.33 - 1.95]

baseline PF			
60-64			
prefrail	1.01<br>[0.72 - 1.41]	1.25<br>[0.84 - 1.87]	0.59<br>[0.32 - 1.07]
frail	0.48<br>[0.19 - 1.18]	3.64*<br>[1.25 - 10.56]	0.45<br>[0.08 - 2.40]
65-80			
prefrail	0.83<br>[0.65 - 1.07]	1.52**<br>[1.12 - 2.06]	0.72<br>[0.48 - 1.08]
frail	0.85<br>[0.49 - 1.47]	1.08<br>[0.51 - 2.26]	1.02<br>[0.49 - 2.14]
⩾80			
prefrail	1.87<br>[0.62 - 5.64]	0.72<br>[0.21 - 2.51]	0.30<br>[0.05 - 2.02]
frail	1.53<br>[0.27 - 8.49]	0.64<br>[0.05 - 8.76]	1.18<br>[0.11 - 13.04]

Note: *** P<0.001, ** P<0.01, * P<0.05. The model had been adjusted for all covariates. Model 1 had been adjusted for the components numbers in the PFP scale at baseline and Model 2 had been adjusted for the baseline levels of loneliness.

Table S5. Standardized estimates of the cross-lagged relationship between change in loneliness and change in physical frailty

	60-64	65-70	⩾70	60-64	65-80	⩾80
Correlation path						
T_1 loneliness ↔ T_1 PF	0.27^{***} (0.022)	0.26^{***} (0.027)	0.27^{***} (0.026)	0.27^{***} (0.022)	0.27^{***} (0.019)	0.25^{**} (0.079)
Δloneliness ↔ ΔPF	0.21^{***} (0.024)	0.20^{***} (0.031)	0.14^{***} (0.029)	0.21^{***} (0.024)	0.17^{***} (0.022)	0.11 (0.086)
Autoregressive path						
T_1 loneliness → Δloneliness	-0.59^{***} (0.020)	-0.60^{***} (0.023)	-0.63^{***} (0.021)	-0.59^{***} (0.020)	-0.62^{***} (0.016)	-0.62^{***} (0.058)
T_1 PF → ΔPF	-0.66^{***} (0.017)	-0.63^{***} (0.021)	-0.61^{***} (0.021)	-0.66^{***} (0.017)	-0.62^{***} (0.015)	-0.59^{***} (0.057)
Cross-lagged path						
T_1 loneliness → ΔPF	0.06^{**} (0.021)	0.04 (0.026)	0.08^{**} (0.025)	0.06^{**} (0.021)	0.06^{**} (0.019)	0.05 (0.069)
T_1 PF → Δloneliness	0.04^{*} (0.022)	0.08^{**} (0.027)	0.04^{+} (0.023)	0.04^{*} (0.022)	0.06^{**} (0.019)	0.02 (0.063)

Note： *** $P<0.001$, ** $P<0.01$, * $P<0.05$, $^{+}$ $P<0.1$.
